# Relationships among microbiota, gastric cancer, and immunotherapy

**DOI:** 10.3389/fmicb.2022.987763

**Published:** 2022-09-12

**Authors:** Yuzhen Li, Xiaona Huang, Desheng Tong, Chenyu Jiang, Xiaodan Zhu, Zhipeng Wei, Tingjie Gong, Chunhui Jin

**Affiliations:** ^1^Department of Oncology, Wuxi Hospital Affiliated to Nanjing University of Chinese Medicine, Wuxi, China; ^2^Key Laboratory of National Health and Family Planning Commission on Parasitic Disease Control and Prevention, Jiangsu Provincial Key Laboratory on Parasite and Vector Control Technology, Jiangsu Institute of Parasitic Diseases, Wuxi, China

**Keywords:** gastric cancer, microbiota, immune checkpoint inhibitors, immune response, PD-L1, CTLA-4

## Abstract

Currently, conventional neoadjuvant therapy or postoperative adjuvant therapy, such as chemotherapy and radiation therapy, can only bring limited survival benefits to gastric cancer (GC). Median survival after palliative chemotherapy is also low, at about 8–10 months. Immunotargeting is a new option for the treatment of GC, but has not been widely replicated. The highly immunosuppressed tumor microenvironment (TME) discounts the efficacy of immunotherapy for GC. Therefore, new strategies are needed to enhance the immune response of the TME. This paper reviewed the relationship between microorganisms and GC, potential links between microorganisms and immunotherapy and research of microorganisms combined immunotherapy.

## Background

Despite its lowering prevalence, gastric cancer (GC) is still plaguing the world. According to the latest data released by International Agency for Research on Cancer (IARC) (Sung et al., 2021), GC has the fifth highest prevalence (1.09 million cases) and the fourth highest mortality (770,000 deaths) in 2020. Most of GC cases, once confirmed, have already entered an advanced stage. Surgical resection remains the primary option for GC, but the associated 5-year survival rate is still less than 60% (Koumarianou et al., 2019). Unfortunately, conventional neoadjuvant therapies or postoperative adjuvant therapies, such as chemotherapy and radiotherapy, can only provide a marginal survival benefit. About 50% of cases develop local recurrence or systemic metastasis after adjuvant treatment, and only 10–15% of the cases have an overall survival (OS) of 5 years. Metastases are primarily treated with palliative chemotherapy, leaving a lower median survival rate of about 8–10 months (Nishikawa et al., 1990, 2019; Alvarez-Manceñido et al., 2021). Among the currently used targeted drugs, only Trastuzumab and Ramucirumab have shown beneficial results in improving OS (Lazăr et al., 2018).

Immunotherapy, which targets the host immune system, has completely changed the landscape of cancer treatment. Blocking immune checkpoints, such as cytotoxic T lymphocyte associated antigen-4 (CTLA-4), programmed cell death protein 1 (PD-1), and its ligands (PD-L1 or B7-H1), have proved effective for several solid cancers (Ansell et al., 2015; Motzer et al., 2015; Ferris et al., 2016; Antonia et al., 2017; Weber et al., 2017). Immunotargeting is promising a new option for the treatment of GC.

By using gene expression data, the Asian Cancer Research Group has described four molecular subtypes of GC, including the subtypes of epithelial-mesenchymal transition (EMT), microsatellite instability (MSI), microsatellite stability (MSS)/TP53+ and MSS/TP53-(The Cancer Genome Atlas Research Network, 2014; Cristescu et al., 2015; Oh et al., 2018). Based on the comprehensive description for its molecular landscape, GC is divided into four subtypes, including encompassing chromosomal instability (CIN), MSI, genome stable (GS), and EBV (The Cancer Genome Atlas Research Network, 2014). Despite the deepened understanding of the molecular subtypes of GC, little is known about the cell-infiltrating characterizations of tumor microenvironment (TME). The efficacy of immunotherapy varies with the immunogenicity of TME as well as the TME heterogeneity and complexity formation (Han et al., 2021). TME contains microorganisms, which can be regulated to enhance the efficacy of immunotherapy (Angelova et al., 2015; Ribas and Wolchok, 2018; Greally et al., 2019).

Here, we review analyses of the microorganisms of different body parts (in and around the stomach, oral and intestinal areas) in GC patients, discuss the potential relationship between microorganisms and immunotherapy, and summarize several current studies on microorganisms combined immunotherapy.

## Relationship between microorganisms and gastric cancer

### Correlation between intragastric and perigastric microbiota and gastric cancer

**FIGURE 1:**
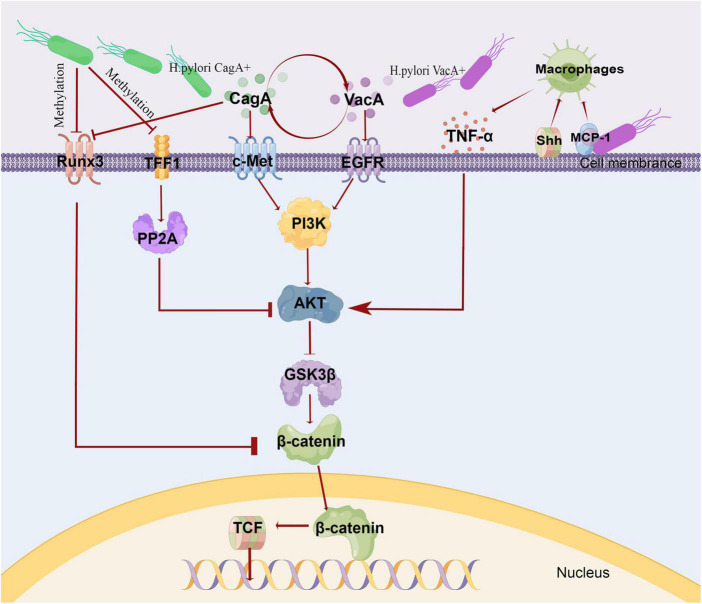
Mechanism of gastric cancer induced by HP. The virulence of HP was expressed through various pathogenic markers, such as CagA, VacA. VacA and CagA could regulate each other. CagA induced c-Met phosphorylation, triggered PI3K/Akt signaling, and caused β-catenin accumulation. VacA induced EGFR phosphorylation and activated PI3K/Akt pathway. In HP infection, the intracellular pathway initiated by EGFR and c-Met converges to PI3K\/AKT-GSK3β−−β catenin. Due to promoter methylation, the Wnt/β-catenin repressor TFF1 and RUNX3 were often down-regulated, and RUNX3 was also associated with CagA. TFF1 inhibited Akt and GSK3β phosphorylation through PP2A, and then reduced β-catenin nuclear translocation and TCF transcription activity. HP infection recruited macrophages *via* MCP-1or Shh. These macrophages produced pro-inflammatory cytokines, such as TNF-α,which could activate Wnt/βcatenin *via* Akt-GSK3β signaling in GC. This fig was made by Figdraw. CagA, cytotoxin-associated gene A; VacA, vacuolating cytotoxin A; TFF1, Trefoil factor 1; PP2A, protein phosphatase 2A; PI3K, phosphatidylinositol 3-kinase; MCP-1, monocyte chemoattractant protein-1; Shh, Sonic Hedgehog.

TME plays a key role in the occurrence, development, and metastasis of cancer. Intragastric and perigastric microbiota have been proven an important part of tumor microenvironment (Ling et al., 2019; Smet et al., 2021). *Helicobacter pylori* (HP) infection, a major risk factor for GC and has been extensively studied. Correa (1992) proposed a multi-step model to elucidate the mechanism of gastric microbiota in GC, which can progress from chronic superficial gastritis to atrophic gastritis, intestinal metaplasia, dysplasia, and eventually to GC; this model proved the contributing role of HP in GC. Later exploration found that The mechanism of GC caused by HP infection may be related to the effect of virulence factors (Amieva and Peek, 2016), and Wnt/β-catenin was a key pathway of GC (Song et al., 2015). HP could up-regulate the Wnt/β-catenin activator c-met (Suzuki et al., 2009; McCracken et al., 2014) and EGFR (Yan et al., 2009; Chaturvedi et al., 2014), down-regulate the Wnt/β-catenin repressor TFF1 (Ito et al., 2008, 2011; Tsang et al., 2010) and RUNX3 (Lefebvre et al., 1996; Park et al., 2000; Tomita et al., 2011). HP could also activate the Wnt/β-catenin pathway by recruiting tumor-associated macrophages (Oguma et al., 2008; Oshima et al., 2011). Importantly, through the Wnt/β-catenin pathway, HP induced the production and expansion of gastric stem cells, which promoted the occurrence and development of GC. The relevant mechanism is shown in Figure 1. Besides, HP infection was also found associated with host genetic susceptibility and interactions with other environmental factors such as smoking and diet (Pereira-Marques et al., 2021).

Apart from HP, there are many other microorganisms in the stomach, and their interactions are necessary in the maintenance of GC, which has been the focus of current research. The main microbiota in the human stomach contain five phyla, including *Proteobacteria*, *Firmicutes*, *Bacteroidetes*, *Actinobacteria*, and *Fusobacteria* (Hsieh et al., 2018; Pereira-Marques et al., 2019). For microbial diversity, different studies have shown opposite results due to differences in sample types, ranking methods, geographical sources, and population environmental exposure. Chen X. et al. (2019) used 16S rRNA gene sequencing and PICRUSt to predict the functional distribution of the microbiota, and constructed a co-occurrence network to analyze the interaction between gastric microbiota. The results showed that compared with the non-cancerous gastric tissue, the richness and diversity of microorganisms increased in cancerous tissue, making a symbiotic network more complicated. Oral bacteria (such as *Streptococcus* and *Fusobacterium*) were enriched in the cancerous tissue, while lactic acid produced bacteria (such as *Lactococcus lactis* and *Lactobacillus brevis*) in adjacent non-cancerous tissue. Another study showed an increasing diversity of gastric mucosa bacteria ranging from atrophic gastritis, intestinal metaplasia, to GC (Gantuya et al., 2020). In addition, Wang L. et al. (2020) found this diversity increased in GC or gastritis stage by using gas chromatography.

Other studies have reported the decrease in microbial richness and diversity in GC patients, especially in those with HP (Ferreira et al., 2018; Liu et al., 2019). Wang et al. (2022) performed metagenomic shotgun sequencing on stomach swab samples from 96 patients with GC, and then conducted metagenomic association analysis between changes in stomach microbiota and HP infection status. It was found that HP became the dominant species after colonization in human stomach, and significantly reduced the α diversity of gastric community. A study in Portugal also showed that as the disease progressed from gastritis to GC, the diversity index gradually decreased and the number of non-HP proteus increased (Ferreira et al., 2018). Compared with patients with chronic gastritis, the number of several bacteria (including *Streptococcus*, *Prevotella*, *Achromobacter*, *Citrobacter*, *Clostridium*, *Rhodococcus*, *Lactobacillus*, and *Phyllobacterium*) was significantly increased in patients with GC (Ferreira et al., 2018). Wang L. et al. (2020) reported that the bacterial richness and diversity in the gastric mucosa gradually decreased across non-atrophic chronic gastritis, intestinal metaplasia, intraepithelial neoplasia, and GC.

The mechanism of microorganisms other than HP affecting the occurrence and development of GCr has also been reported. Based on the map obtained from the 16S rRNA gene sequence, Ferreira et al. (2018) completely reconstructed a metagenome showing that compared with chronic gastritis, GC occurred with enhanced function of nitrate reductase, which degrades nitrate into nitrite, and nitrite into nitric oxide. *Fourgenera of citrobacter, Achromobacter*, *Clostridium*, and *Phyllobacterium* have been identified as the major contributors. Interestingly, this result is consistent with a follow-up study in Taiwan that evaluated the effect of subtotal gastrectomy as a treatment for early GC (Tseng et al., 2016). These data suggest that the gastric microbiome has the potential to produce carcinogenic nitroso compounds during the development of cancer. Furthermore, toxic metabolites and inflammation by abnormal microbiota products may directly damage host cells or interfere with host signaling pathways engaged in cell turnover and survival, thereby increasing the risk of gastric malignant transformation (Pereira-Marques et al., 2019).

Given that microbiological disorder is a factor of GC, identifying related bacterial species is of great clinical significance. It is undeniable that HP, a common risk factor for GC (Yu et al., 2017), colonizes to disrupt the structure of microbiota. Here, through several microbiological studies on Chinese GC population (Table 1; Yu et al., 2017; Hsieh et al., 2018; Chen X. et al., 2019; Wang L. et al., 2020; Wang Z. et al., 2020; Dai et al., 2021; Wu et al., 2021; Zhang et al., 2022b), we found that the enrichment of *Clostridium*, *Bacteroid*, and *Lactobacillus* was positively correlated with the occurrence and development of GC. *Clostridium* has also been found to be associated with poor prognosis of GC (Hsieh et al., 2018; Boehm et al., 2020; Nie et al., 2021). In addition, ROS produced by lactic acid bacteria can damage DNA, promote tumor growth and metastasis, and inhibit tumor apoptosis by promoting the production of N-nitroso compounds (NOCs) (Jones et al., 2012; Ling et al., 2019). Furthermore, a recent animal study suggested that the increase of *Lactobacillus* richness accelerated the progress of GC, turning it a potential biomarker (Dai et al., 2021). A study also revealed that *Candida albicans* initiated GC progress by reducing the diversity and abundance of microbes in the stomach (Zhong et al., 2021). Zhang et al. (2022a) observed a dysregulation of fungal flora on gastric mucosa between the GC group and the normal group, and the abundance of some taxa in the GC group was higher than that in the normal group. Lefse analysis found that *Solicocozyma* was differentially enriched at the genus level in GC group and was considered as a gastric fungal marker, and functional predictions suggested that the positive expression of *Solicocozyma* in tumors was associated with amino acid and carbohydrate-related metabolic pathways in GC.

**TABLE 1:** Genera depleted and/or enriched in cancer vs. non-cancer.

N	Diagnosis (sample size)	Country	Genera depleted and/or enriched in cancer vs. non-cancer	References
160	GC(80) VS. Non-cancer(80)	China, Mexico	*↑Bacteroidetes, Firmicutes, Fusobacteria, Spirochaetes* ↓*Proteobacteria*	Yu et al., 2017
27	GC(11) vs. Non-cancer(16)	China	*↑Clostridum, Fusobacteruim, Lactobacillus*	Hsieh et al., 2018
124	GC(62) vs. Non-cancer(62)	China	*↑Peptostreptococcus, Streptococcus, and Fusobacterium*	Chen X. et al., 2019
132	GC(29) VS. IN(25) VS. IM(27) VS. CG(21) VS. HC(30)	China	*↑Actinobacteria, Bacteriodes, Firmicutes, Fusobacteria, SR1, and TM7* ↓*phyla Armatimonadetes, Chloroflexi, Elusimicrobia, Nitrospirae, Planctomycetes, Verrucomicrobia, and WS3*	Wang L. et al., 2020
120	GC(60) vs. CG(60)	China	*↑Novosphingobium, Ralstonia*, *Ochrobactrum, Anoxybacillus, and Pseudoxanthomonas*	Wang Z. et al., 2020
50	GC(18) vs. SG(32)	China	*↑Dialister, Lactobacillus, Rhodococcus, Sediminibacterium*	Wu et al., 2021
74	GC(37) vs. Non-cancer(37)	China	*↑Lactobacillus, Streptococcus, Acinetobacter, Prevotella, Sphingomonos, Bacteroides, Fusobacterium, Comamonas, Empedobacter, Faecalibacterium*	Dai et al., 2021
52	GC(22) vs. Non-cancer(30)	China	*↑Proteobacteria, Prevotella_9, Streptococcus, and Lactobacillus*	Zhang et al., 2022b

IN, intraepithelial neoplasia; IM, intestinal metaplasia; CG, chronic gastritis; HC, :healthy controls; SG, superfcial gastritis; ↑, GC enriched; ↓, GC depleted.

### Correlation of oral microbiota with gastric cancer

Mounting evidence shows that human oral microbe is related to the development of digestive system cancer (Tuominen and Rautava, 2021). A recent study by Coker et al. (2018) examined the bacterial taxonomy in patients with superficial gastritis, atrophic gastritis, intestinal metaplasia, and GC, using 16S RNA sequencing. They found that, the oral microbiota were more abundant in GC specimens than in benign and precancerous specimens, especially digestive *Streptococcus*, *Clostridium*, *Dialysis bacteria*, P*roteobacteria*, and vascular *Streptococcus*. In addition, bacterial network analysis showed that the interaction of these oral microorganisms with other bacteria in the gastric mucosa was more intensive. A study in the United States compared the periodontal pathogen abundance in saliva and dental plaque samples from 35 patients with pathological changes before GC and 70 controls. They found that people predisposed to GC has the enriched *T. forsythia*, *T. Denticola*, and *A. actinomycetemcomitans* in their oral microorganisms (Sun et al., 2017). People prone to GC also has different oral microbial compositions. For example, the pyrosequencing of 16S rRNA genes in the tongue coating microbiome of 57 patients with newly diagnosed GC and 80 healthy controls showed higher relative abundance of *Firmicutes* and lower relative abundance of *Bacteroidetes* in the oral microbe of people with GC. At the genus level, GC patients have a higher abundance of *Streptomyces* (Wu et al., 2018). A prospective study of oral microbiome and GC risk demonstrated that people with high risks of GC have the decreased microbial abundance of *Tenericutes*, *M. Orale*, *E. Yurii*, and *Cutibacterium*, and increased abundance of *Betaproteobacteria*, *Neisseriales*, *Neisseriaceae*, *N. mucosa*, and *P. pleuritidis* (Yang et al., 2022b). The above studies have suggested that oral microbiota may be an important factor in maintaining GC, and the detection of oral microbiota may help in early diagnosis and screening of GC. Therefore, Sun et al. (2018) examined the total bacterial spectrum of saliva and plaque samples from 50 subjects (including 37 patients with GC and 13 controls), using high-throughput sequencing technology. The Venn diagrams and clusters generated from the data suggested that the oral bacteria of GC patients were more intricate. According to the characteristics of oral microbiome of GC patients, a scoring system was designed to screen GC, with a sensitivity of 97%.

Studies have found that oral microorganisms can cause excessive inflammatory response, especially *Clostridium, porphyromonas*, and *Prevotella*, which can cause oncogene activation, mutation, DNA damage, cell cycle arrest, cell proliferation, tumor invasion, migration, metastasis, and angiogenesis (Ahn et al., 2012; Szkaradkiewicz and Karpinski, 2013). They can promote anti-apoptosis of cancer cells, such as *Mycobacterium nucleonucleus* (Haura et al., 2005) and *Pseudomonas gingivalis* (Michaud, 2013), which ultimately inhibits apoptosis by activating anti-apoptotic signaling pathway and inhibiting pro-apoptotic pathway, leading to cancer growth. They can also produce carcinogens, eventually leading to GC (Sun et al., 2020).

### Correlation between gut microbiota and gastric cancer

The human gut microbiota (GM), with more than 100 trillion microbial cells, are a symbiotic system in the host and a key regulator in host metabolism. Therefore, significant changes in their composition and function are associated with many diseases, including cancer (McQuade et al., 2019). In childhood, *Bifidobacterium* initially dominates GM to resist the inflammatory pregastrointestinal environment, which is typical in this stage of life (Arboleya et al., 2016). In adulthood, *Firmicutes* and *Bacteroidetes* make up 90% of GM, with *Actinomycetes*, *Proteus*, *Fusobacterium*, and *Verrucomicrobia* composing the remaining 10%. They synthesize short chain fatty acids (SCFA) that enable the host to digest plant polysaccharides and extract energy from the diet (Cătoi et al., 2020). As the host ages, bacterial biodiversity gradually drops, pathogens (such as clostridium) flourish, and butyric acid-producing bacteria undergo rearrangement (Brandi et al., 2017).

GM is associated with GC (Vogelmann and Amieva, 2007; Chen D. et al., 2019), and HP plays an important role in this association. Wang D. et al. (2019) sequenced 313 fecal samples using macrogenomic shotgun method, finding that HP infection was associated with changes in the composition and function of intestinal microbiota in Chinese people. Scher et al. (2013) found that *P. copri* was significantly enriched in HP positive individuals, and also very active in the pro-inflammatory gastrointestinal environment, which could further increase the inflammation level. Some researchers also found that *P. copri* was related to immune rheumatoid arthritis (Zhang et al., 2015; Pianta et al., 2017). Therefore, *P. copri* may be related to the changes of intestinal immune environment. Previous multi-time-point follow-up studies reported that fecal microbial diversity decreased significantly in a short term after eradication of HP for 1 or 2 weeks, but then recovered slowly (Yap et al., 2016; Liou et al., 2019). This is consistent with the findings of Guo et al., 2020 who also found that successful eradication of HP had more beneficial effects on the GM than failed treatments, such as probiotic enrichment and down-regulation of resistance mechanisms (Guo et al., 2020). Lertpiriyapong et al. (2014) found that the co-infection of HP and three other GM (*Clostridium ASF356*, *Lactobacillus ASF361*, and *Bacillus ASF519*) led to the formation of GC in transgenic mice with germ-free human gastrin overexpression (INs-GAS mice). The above evidence suggested that there was interactions between HP and GM, and HP colonization is a risk factor for GC development, so GM may play an important role in the maintenance of GC.

Other studies have found that the abundance of some GM in patients with GC is different, but whether it significantly affects the occurrence and development of GC is still controversial. A study of fecal samples from 10 patients with gastric adenocarcinoma showed that *Bacteroides* was the most abundant genus in all samples, followed by *Blautia* (7.1%), *Veillonella* (6.4%), and *Sartrella* (8.2%) (Wang F. et al., 2019). Yet another study analyzing the diversity and composition of GM in the fecal samples of 20 GC patients and 22 healthy controls revealed that the GC group had higher contents of *Shigella*, *Clostridium perfringens*, and *Clostridium*, and a lower content of *Bacteroides* (Liang et al., 2019). *Bifidobacterium* is also an important part of GM. It is worth mentioning that Sivan et al. (2015) and Sarhadi et al. (2021) found that *Bifidobacterium* was less abundant in diffuse gastric adenocarcinoma; meanwhile, the gastric adenocarcinoma was less infiltrated by immune cells and more aggressive. This study also showed that oral *Bifidobacteriu* controlled tumor growth in mice by increasing T cell accumulation in the TME and enhancing the efficacy of programmed cell death protein 1 (PD-L1) specific antibody therapy (Sivan et al., 2015). Thus, intestinal *Bifidobacterium* acts on tumor growth through mediating host immunity, and *Bifidobacterium* supplements may have a beneficial effect on cancer patients.

The structure of GM in patients with GC is affected by different tumor types, regardless of surgery or treatment. Recently, a study in Finland found that a high abundance of *Enterobacteriaceae* was a common GM feature of all GC subtypes. Patients with diffuse gastrointestinal stromal tumor and diffuse gastric adenocarcinoma exhibited lower intestinal microbiota diversity, which might be related to the stronger aggressiveness of higher stages of tumors (Sarhadi et al., 2021). Radical gastrectomy also has a significant impact on the composition of intestinal microbiota. Wang F. et al. (2019) and Erawijantari et al. (2020) found that the relative abundances of aerobic bacteria (*Streptococcus* and *Enterococcus*), facultative anaerobe (*Escherichia coli*, *Enterobacter*, and *Streptococcus*) and oral microbiota in postoperative patients were higher than those in the control group. Chemotherapy also has an impact. Zhang et al. (2021) found that chemotherapy reduced the abundances of some intestinal bacteria in GC patients, but most of these bacteria had shown enrichment in gas chromatograph.

Therefore, there is no unified conclusion on the composition of GM in patients with GC. The composition of GM is the result of multiple factors, which requires studies with a large sample size for statistics. On the mechanism of GM affecting the occurrence and development of GC, it has been reported that the intestinal microbiome may pose carcinogenic effects by inducing oxidative stress, genotoxicity, host immune response dysfunction, and chronic inflammation (Weng et al., 2019).

## Effects of microbiota on immunotherapy for gastric cancer

### Gastric tumor microenvironment and immune response

In humans, there are two types of adaptive immune response: humoral immunity mediated by B cell antibody and cellular immunity mediated by T cells, including CD8 (TC) and CD4 (th) cells. The immune system keeps the body homeostasis by defending itself against infections and diseases caused by bacteria, viruses, fungi and parasites. However, the immune system can also constantly detect and remove precancerous cells to prevent the progression to malignancy (Marzagalli et al., 2019). Nevertheless, to evade immune surveillance, tumor cells release proteins (such as CTLA-4, PD-1, and its ligand PD-L1) that negatively regulate the immune response, so using antibodies to directly block those negative immune regulators (checkpoints) has proved to be an important strategy to enhance immunity against cancer. At present, three types of immune checkpoint inhibitors (ICIs), namely, anti-CTLA-4, PD-1, and PD-L1, have been developed and proved effective for a variety of malignant cancers (Olnes and Martinson, 2021).

Although immunotherapy is advancing rapidly, it is still not as effective as conventional chemotherapy because tumor cells can induce an immunosuppressive microenvironment. GC can be subdivided into immunogenicity subtypes and immune tolerance subtypes. Ren et al. (2021) have separated GC into three types according to the infiltration of 34 immune cells. Clusters 1 and 2 are filled with immune cells, antigen-presenting cells, and immunomodulatory molecules, suggesting a preexisting antitumor immune response. In contrast, Cluster 3 has fewer immune cells, MHC molecules, and immunomodulatory molecules. The researchers defined Cluster 1 and 2 as hot tumors and Cluster 3 as cold tumors. Hot tumors can activate immune function-related pathways. However, cold tumors may promote mutagenesis by inducing changes to epigenome through genomic instability and transcriptional changes. In particular, CLDN3 is a key immunosuppressive modulator, and targeting CLDN3 may program cold into hot tumors and improve the efficacy of tumor immunotherapy (Ren et al., 2021).

### Microbiome influence the immunotherapeutic response

Apart from the immune microenvironment of tumor, the microbiota also plays a crucial role in immunotherapy (Olnes and Martinson, 2021). In fact, the association among HP, immune microenvironment (TME) and malignancy has long been recognized in GC (Deng et al., 2021). It has been demonstrated in the studies of Geng et al. (2022), which proposed a molecular prognostic feature specifically designed for GC patients with HP infection. According to the review by Oster et al. (2022), HP infection has an adverse effect on immunotherapy for cancer, but there is no evidence of immunotherapy for GC. A recent retrospective study was the first to reveal that patients with HP infection had a shorter OS and PFS than those in the negative group (Che et al., 2022). Therefore, whether HP can affect the efficacy of immunotherapy in GC patients is still worth exploring and studying.

**FIGURE 2:**
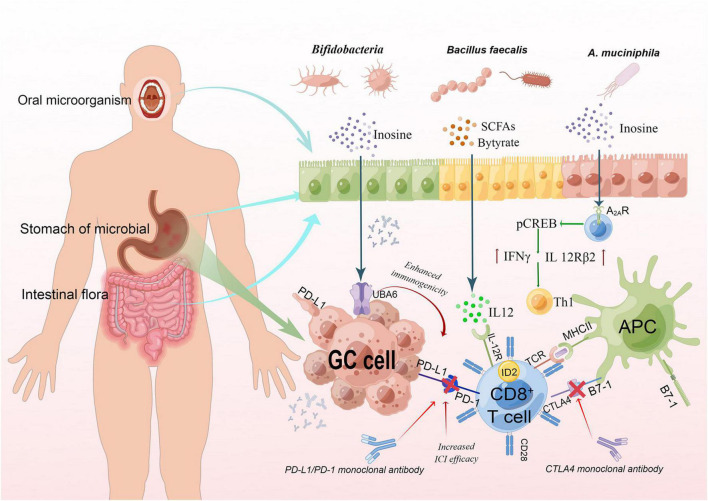
The relationship between microbiota and immunosuppressive of GC. The interaction between TCR and peptide-MCHII on APC and a second costimulatory signal mediated by CD28 can activate T cells, while CTLA4 binds to B7-1 to inhibit the activation of T cells. Binding of PD-L1 on GC cells to PD-I on T cells can also inhibit T cell activation, so blocking the binding can activate T cells. Gastrointestinal bacteria were found to act synergistically. Butyric can induce CD8 + T cells to express ID2 through IL-12 signal transduction, and directly enhance the anti-tumor cytotoxicity of CD8 + T cells. Inosine could enhance ICI efficacy by acting on A2AR on T lymphocytes. It stimulates the phosphorylation of cAMP response element-binding protein (pCREB) through the inosine-A2AR-cAMP-PKA signaling pathway, which can upregulate IL12Rβ2 and IFNγ transcription and promote Th1-cell differentiation and accumulation in the TME. This fig was made by Figdraw. TCR, T cell receptor; MHC, major histocompatibility complex; APC, antigen-presenting cells.

For GM, DNA sequencing analysis of fecal samples taken before treatment with PD1 inhibitors revealed different microbiota compositions between responders and non-responders, suggesting a link between GM composition and subsequent treatment response. The mouse model reconstructed with fecal isolates from responders had greater gains from checkpoint blockade therapy than that with fecal samples from non-responders (Matson et al., 2018), which further confirmed the link between microbiota and the efficacy of immunotherapy. The relationship between microbiota and immunosuppressive of GC is provided in Figure 2. Preliminary evidence from mice studies suggests that specific microorganisms help ICIs related immune therapy. For example, the control of tumor growth by oral bifidobacteria alone was the same as PD-L1 specific antibody therapy (Weng et al., 2019). Similarly, bifidobacterium can also enhance the efficacy of cancer immunotherapy through CTLA (Vétizou et al., 2015). In terms of clinical studies, Peng Z. et al. (2020) investigated the characteristics of GM associated with clinical response to anti-PD1/PD-L1 immunotherapy in a cohort of patients with gastrointestinal cancer (19 colorectal cancer, 23 GC, 14 esophageal cancer, and 18 other gastrointestinal cancer types). They found that in this small mixed group, there was no significant differences in GM diversity between responders and non-responders regardless of cancer type, but the abundance of *Bacteroides* appeared to be higher in non-responders compared with responders for each cancer type. Other studies have found that for PD-1/PD-L1, differences in immunotherapy responses are linked to the composition of intestinal microbiota. Especially, compared with non-respondents, *A. muciniphila was* proved to be more abundant in PD-1 treatment respondents (Routy et al., 2018). In short, host microbiota can be modulated to enhance the host response and reduce the side effects of immunotherapy (Vétizou et al., 2015; Cramer and Bresalier, 2017; Qiu et al., 2021).

### Gut microbiota metabolites affect immunotherapy response

Normal GM synthesize a variety of immunomodulatory compounds and metabolites, such as SCFA, propionate, acetate, and butyrate, as well as secondary bile acids and ubiquitous bacterial fermentation products (Huang et al., 2021). These bioactive agents can regulate the sizes, metabolic processes and functions of receptors on immune cells, which may bring health benefits to the host (Belkaid and Harrison, 2017). It was found that factors influencing the effective response of PD-1/PD-L1 included the infiltration and localization of early tumor-infiltrating lymphocyte (TIL), activation level of TIL and effects of tumor cell mutation (Kansy et al., 2017; Hellmann et al., 2018; Gao and Chen, 2021). GM can promote anti-tumor immune response by several mechanisms, including triggering T cell responses to the bacterial antigens. Bacterial antigens can cross-react with tumor antigens, or recognize receptors through mediating immunogenicity and anti-inflammatory effects, or recognize tumor specific antigens through mediating the effect of small metabolites on the host (Zitvogel et al., 2018).

SCFA can prevent cancer by regulating cell cycle through Akt/mTOR and MEK/ERK signaling pathways, apoptosis by transcription factors (NF-kB), and immune responses by inhibiting HDACs activity, DNA methylation, histone phosphorylation and methylation (Orchel et al., 2005; Feng and Qiu, 2018). Among them, butyrate plays an important role in human body and has strong anti-cancer activity (Singh et al., 2014; Feng and Qiu, 2018). Butyrate, mainly produced by *Bacillus faecalis*, enhances not only gastrointestinal immunity and maintain the integrity of the intestinal barrier (Fu et al., 2019; Ratajczak et al., 2019), but also carcinogenesis by increasing the proliferation of abnormal epithelial cells (Matthews et al., 2007; Naseem et al., 2018). Recently, Oster et al. (2022) found that the GM metabolite butyrate improved the efficacy of oxaliplatin by regulating the function of CD8 + T cells in the TME.

Inosine, another normal metabolite of intestinal flora, is mainly produced by Ackermann mucilagium and b. pseudolongum, and can activate immune cells and stimulate metabolism in physiological state. Inosine can reprogram TME and improve the response to ICI treatment (Correa, 1992; Lioux et al., 2016). It can also enhance the response to immunotherapy by promoting the immunogenicity of tumor cells and the activation of immune cells (Lu et al., 2022). Inosine significantly enhances the ability of tumor cells to present tumor antigens, so that cytotoxic immune cells can easily recognize and kill tumor cells, thus achieving antitumor effects (Bird, 2020). Mechanistic studies showed that inosine was associated with significantly increased activation of IFN-γ and TNF-α signaling pathways in tumor cells. IFN-γ activates cytotoxicity of tumor-specific T cells and NK cells by promoting the release of perforin and granzyme to promote inosine-mediated antitumor effects (Harjes, 2020). Inosine also activates macrophages to stimulate B-lymphocyte differentiation and antibody production, enhancing anti-tumor immune response and plant hemagglutinin (PHA)-mediated immune response (Shinohara and Tsukimoto, 2018; Wang T. et al., 2020).

## Research methods of microbial combined immunotherapy

At present, to enhance the efficacy of immunotherapy, microbiota are mainly used in fecal bacteria transplantation therapy, biological agent therapy, nanotechnology therapy, etc. Most of these techniques are applied in solid tumors other than GC. Whether they are also effective in GC is unknown, but it can provide ideas for future research.

Fecal microbial transplants (FMT) can fight tumors by “repairing” intestinal microbiota. Healthy intestinal microbiota can be transplanted to reconstruct the patient’s intestinal microbiota and increase the proportion of regulatory T cells in the colonic mucosa, and has great potential in reducing the side effects of cancer immunotherapy (Wang et al., 2018; Xu et al., 2021). Studies showed that FMT regulated tumor-related intestinal microbiome and immunity, and could be used as a mainstay therapy for pancreatic cancer (Chandra and McAllister, 2021). Frankel et al. (2017) demonstrated the presence of *Bacteroidetes* in melanoma patients who responded to ICIs. They also found that the antibodies used in cancer immunotherapy were associated with the types of bacteria in responders. The intestinal microbiota were rich in filamentous *Haldermania*, *Enterococcus faecalis*, and *Bacteroides polyformis* among cases who responded to navuliuzumab, but rich in *Doloides* among those responded to paboliuzumab. Therefore, the combination of FMT with chemotherapy and immunotherapy provides a new idea for the treatment of GC. A clinical study is being conducted to prove the improvement of FMT capsules in the anti-PD-1 efficacy for digestive system cancer (No. nct04130763).

Nanotechnology can be employed to target the microbiota to treat cancer (Song et al., 2019). In the treatment of GC, an engineered nanoparticle encapsulating antibiotics can target HP on the gastric membrane and release antibiotics in the target area (Angsantikul et al., 2018). This prophylactic strategy relies on nanotechnology to selectively kill cancer-causing microbes before tumor formation (Inamura, 2021). A recent animal study also found that HP-infected mice responded less to CTLA-4 alone or its combination with anti-PD-L1 than uninfected mice, which was not associated with HP-induced intestinal microflora changes (Oster et al., 2021). Therefore, anti-HP infection is critical in the treatment of GC.

Biologic therapy has been experimented in animal studies of melanoma. Researchers evaluated the function of microorganisms in enhancing the immune defense against tumor in the mice with solid tumor after oral administration of *Bifidobacterium longum* and *Bifidobacterium brevis* cocktails. It was found that tumor growth was better controlled compared with that in untreated mice (Sivan et al., 2015), suggesting that *Bifidobacterium* cocktails can cooperate with immune checkpoint inhibitors to activate anti-tumor immunity. The application of *Bifidobacterium* could be extended to other types of cancer. The effective treatments for other types of cancer may also be applied to GC.

Researchers should focus on a better understanding of gut microbe interaction and how they interact with the host, in order to improve the success rate of the probiotic or FMT treatment, especially future personalized cancer treatments based on microbial cocktails (Oster et al., 2021).

## Expectation

Microbes, especially HP, have been extensively studied in relation to the occurrence and development of GC (Alarcón et al., 2017; Matsuzaki et al., 2021; Yang et al., 2022a). However, except HP, no new bacteria has been widely recognized as a high risk factor for GC (Pereira-Marques et al., 2021). Genetic mutations such as CDH1 and TP53, lifestyle (including smoking, overweight, low fruit and vegetable consumption, as well as high intake of salt, nitrates, and preserved food) were also associated with an increased GC risk (Yang et al., 1990; Pharoah et al., 2001). Multiple studies have shown that the use of microbiota, especially specific bacteria, can provide new microbial markers for cancer prevention, diagnosis and treatment (Peng C. et al., 2020). This article reviews several bacteria that may be potential biomarkers, but larger and more comprehensive studies are needed to confirm their feasibility.

New treatment options are always designed to prolong the survival of patients, or even cure the tumor. Despite refreshment in GC treatment strategies from chemotherapy, radiotherapy, targeted therapy to immunotherapy over hundreds of years, the mortality rate remains high. Over the past decade, immune checkpoint inhibitors, such as PD-1, PD-L1, and anti-angiogenic monoclonal antibodies, have revolutionized the result of advanced cancer (Peng C. et al., 2020). Microbiota participate in immune regulation through a variety of signal pathways, thereby enhancing the efficacy of tumor immunotherapy and intoxicating drugs. Tools based on bioinformatics, big data and artificial intelligence may be invented to realize precision GC treatment in the future. Even in the absence of a cure for cancer, solutions using microbes to achieve sustainable and long-term disease control are likely to exist to prolong quality of life and meet life needs in the future. This may help to turn an incurable disease into a chronic but manageable one. Better understanding of the internal environmental balance between microbial and cancer systems may ultimately contribute to a long and healthy life (Xavier et al., 2020).

## Author contributions

CJ and YL conceived and designed the study. YL, XH, CJ, XZ, ZW, and TG were responsible for the collection, extraction, and analysis of the literature. YL and XH were responsible for writing the manuscript. DT designed and draw the pictures. CJ polished the English language. All authors and participants reviewed the manuscript and reached an agreement to approve the final manuscript.

## References

[B1] Ahn J. Chen C. Hayes R. (2012). Oral microbiome and oral and gastrointestinal cancer risk. *CCC* 23 399–404. 10.1007/s10552-011-9892-7 22271008 PMC3767140

[B2] Alarcón T. Llorca L. Perez-Perez G. (2017). Impact of the Microbiota and Gastric Disease Development by Helicobacter pylori. *Curr. Topics Microbiol. Immunol.* 400 253–275. 10.1007/978-3-319-50520-6_11 28124157

[B3] Alvarez-Manceñido F. Jimenez-Fonseca P. Carmona-Bayonas A. Arrazubi V. Hernandez R. Cano J. (2021). Is advanced esophageal adenocarcinoma a distinct entity from intestinal subtype gastric cancer? Data from the AGAMENON-SEOM Registry. *Gastric Cancer* 24 926–936. 10.1007/s10120-021-01169-6 33651195

[B4] Amieva M. Peek R. (2016). Pathobiology of Helicobacter pylori-Induced Gastric Cancer. *Gastroenterology* 150 64–78. 10.1053/j.gastro.2015.09.004 26385073 PMC4691563

[B5] Angelova M. Charoentong P. Hackl H. Fischer M. Snajder R. Krogsdam A. (2015). Characterization of the immunophenotypes and antigenomes of colorectal cancers reveals distinct tumor escape mechanisms and novel targets for immunotherapy. *Genome Biol.* 16:64. 10.1186/s13059-015-0620-6 25853550 PMC4377852

[B6] Angsantikul P. Thamphiwatana S. Zhang Q. Spiekermann K. Zhuang J. Fang R. (2018). *Helicobacter pylori* Coating nanoparticles with gastric epithelial cell membrane for targeted antibiotic delivery against infection. *Adv. Ther.* 1:1800016. 10.1002/adtp.201800016 30320205 PMC6176867

[B7] Ansell S. Lesokhin A. Borrello I. Halwani A. Scott E. Gutierrez M. (2015). PD-1 blockade with nivolumab in relapsed or refractory Hodgkin’s lymphoma. *N. Eng. J. Med.* 372 311–319. 10.1056/NEJMoa1411087 25482239 PMC4348009

[B8] Antonia S. Villegas A. Daniel D. Vicente D. Murakami S. Hui R. (2017). Durvalumab after Chemoradiotherapy in Stage III Non-Small-Cell Lung Cancer. *N. Eng. J. Med.* 377 1919–1929. 10.1056/NEJMoa1709937 28885881

[B9] Arboleya S. Watkins C. Stanton C. Ross R. (2016). Gut Bifidobacteria populations in human health and aging. *Front. Microbiol.* 7:1204. 10.3389/fmicb.2016.01204 27594848 PMC4990546

[B10] Belkaid Y. Harrison O. (2017). Homeostatic Immunity and the Microbiota. *Immunity* 46 562–576. 10.1016/j.immuni.2017.04.008 28423337 PMC5604871

[B11] Bird L. (2020). Microbial metabolite boosts immunotherapy. *Nat. Rev. Immunol.* 20 648–649. 10.1038/s41577-020-00465-z 33024282

[B12] Boehm E. Thon C. Kupcinskas J. Steponaitiene R. Skieceviciene J. Canbay A. (2020). Fusobacterium nucleatum is associated with worse prognosis in Lauren’s diffuse type gastric cancer patients. *Sci. Rep.* 10:16240. 10.1038/s41598-020-73448-8 33004953 PMC7530997

[B13] Brandi G. De Lorenzo S. Candela M. Pantaleo M. Bellentani S. Tovoli F. (2017). Microbiota, NASH, HCC and the potential role of probiotics. *Carcinogenesis* 38 231–240. 10.1093/carcin/bgx007 28426878

[B14] Chandra V. McAllister F. (2021). Therapeutic potential of microbial modulation in pancreatic cancer. *Gut* 70:319807. 10.1136/gutjnl-2019-319807 33906958 PMC8292583

[B15] Chaturvedi R. Asim M. Piazuelo M. Yan F. Barry D. Sierra J. (2014). Activation of EGFR and ERBB2 by Helicobacter pylori results in survival of gastric epithelial cells with DNA damage. *Gastroenterology* 146 1739-51.e14. 10.1053/j.gastro.2014.02.005 24530706 PMC4035375

[B16] Che H. Xiong Q. Ma J. Chen S. Wu H. Xu H. (2022). Association of Helicobacter pylori infection with survival outcomes in advanced gastric cancer patients treated with immune checkpoint inhibitors. *Res. Square* [Preprint]. 10.1186/s12885-022-10004-9 35986342 PMC9389789

[B17] Chen D. Wu J. Jin D. Wang B. Cao H. (2019). Fecal microbiota transplantation in cancer management: Current status and perspectives. *Int. J. Cancer* 145 2021–2031. 10.1002/ijc.32003 30458058 PMC6767494

[B18] Chen X. Wang A. Chu A. Gong Y. Yuan Y. (2019). Mucosa-associated microbiota in gastric cancer tissues compared with non-cancer tissues. *Front. Microbiol.* 10:1261. 10.3389/fmicb.2019.01261 31231345 PMC6560205

[B19] Coker O. Dai Z. Nie Y. Zhao G. Cao L. Nakatsu G. (2018). Mucosal microbiome dysbiosis in gastric carcinogenesis. *Gut* 67 1024–1032. 10.1136/gutjnl-2017-314281 28765474 PMC5969346

[B20] Correa P. (1992). Human gastric carcinogenesis: a multistep and multifactorial process–first american cancer society award lecture on cancer epidemiology and prevention. *Cancer Res.* 52 6735–6740. 1458460

[B21] Cramer P. Bresalier R. (2017). Gastrointestinal and hepatic complications of immune checkpoint inhibitors. *Curr. Gastroenterol. Rep.* 19:3. 10.1007/s11894-017-0540-6 28124291

[B22] Cristescu R. Lee J. Nebozhyn M. Kim K. Ting J. Wong S. (2015). Molecular analysis of gastric cancer identifies subtypes associated with distinct clinical outcomes. *Nat. Med.* 21 449–456. 10.1038/nm.3850 25894828

[B23] Cătoi A. Corina A. Katsiki N. Vodnar D. Andreicuţ A. Stoian A. (2020). Gut microbiota and aging-A focus on centenarians. *Biochim. Biophys. Acta Mol. Basis Disease* 1866:165765. 10.1016/j.bbadis.2020.165765 32169505

[B24] Dai D. Yang Y. Yu J. Dang T. Qin W. Teng L. (2021). Interactions between gastric microbiota and metabolites in gastric cancer. *Cell Death Dis.* 12:1104. 10.1038/s41419-021-04396-y 34819503 PMC8613192

[B25] Deng Y. Su W. Zhu J. Ji H. Zhou X. Geng J. (2021). Helicobacter pylori infection disturbs the tumor immune microenvironment and is associated with a discrepant prognosis in gastric de novo diffuse large B-cell lymphoma. *J. Immunother. Cancer* 9:e002947. 10.1136/jitc-2021-002947 34645670 PMC8515460

[B26] Erawijantari P. Mizutani S. Shiroma H. Shiba S. Nakajima T. Sakamoto T. (2020). Influence of gastrectomy for gastric cancer treatment on faecal microbiome and metabolome profiles. *Gut* 69 1404–1415. 10.1136/gutjnl-2019-319188 31953253 PMC7398469

[B27] Feng F. Qiu H. (2018). Effects of Artesunate on chondrocyte proliferation, apoptosis and autophagy through the PI3K/AKT/mTOR signaling pathway in rat models with rheumatoid arthritis. *Biomed. Pharmacother. Biomed. Pharmacother.* 102 1209–1220. 10.1016/j.biopha.2018.03.142 29710540

[B28] Ferreira R. Pereira-Marques J. Pinto-Ribeiro I. Costa J. Carneiro F. Machado J. (2018). Gastric microbial community profiling reveals a dysbiotic cancer-associated microbiota. *Gut* 67 226–236. 10.1136/gutjnl-2017-314205 29102920 PMC5868293

[B29] Ferris R. Blumenschein G. Fayette J. Guigay J. Colevas A. Licitra L. (2016). Nivolumab for Recurrent Squamous-Cell Carcinoma of the Head and Neck. *N. Eng. J. Med.* 375 1856–1867. 10.1056/NEJMoa1602252 27718784 PMC5564292

[B30] Frankel A. Coughlin L. Kim J. Froehlich T. Xie Y. Frenkel E. (2017). Metagenomic shotgun sequencing and unbiased metabolomic profiling identify specific human gut microbiota and metabolites associated with immune checkpoint therapy efficacy in melanoma patients. *Neoplasia* 19 848–855. 10.1016/j.neo.2017.08.004 28923537 PMC5602478

[B31] Fu X. Liu Z. Zhu C. Mou H. Kong Q. (2019). Nondigestible carbohydrates, butyrate, and butyrate-producing bacteria. *Crit. Rev. Food Sci. Nutr.* 59 S130–S152. 10.1080/10408398.2018.1542587 30580556

[B32] Gantuya B. El Serag H. Matsumoto T. Ajami N. Uchida T. Oyuntsetseg K. (2020). Gastric mucosal microbiota in a Mongolian population with gastric cancer and precursor conditions. *Aliment. Pharmacol. Ther.* 51 770–780. 10.1111/apt.15675 32133670 PMC8761497

[B33] Gao L. Chen Y. (2021). Autophagy controls programmed death-ligand 1 expression on cancer cells (Review). *Biomed. Rep.* 15:84. 10.3892/br.2021.1460 34512972 PMC8411486

[B34] Geng H. Dong Z. Zhang L. Yang C. Li T. Lin Y. (2022). An immune signature for risk stratification and therapeutic prediction in *Helicobacter pylori*-infected gastric cancer. *Cancers* 14:3276. 10.3390/cancers14133276 35805047 PMC9265823

[B35] Greally M. Chou J. Chatila W. Margolis M. Capanu M. Hechtman J. (2019). Clinical and Molecular Predictors of Response to Immune Checkpoint Inhibitors in Patients with Advanced Esophagogastric Cancer. *Clin. Cancer Res.* 25 6160–6169. 10.1158/1078-0432.CCR-18-3603 31337644 PMC6905384

[B36] Guo Y. Zhang Y. Gerhard M. Gao J. Mejias-Luque R. Zhang L. (2020). Helicobacter pyloriEffect of on gastrointestinal microbiota: a population-based study in Linqu, a high-risk area of gastric cancer. *Gut* 69 1598–1607. 10.1136/gutjnl-2019-319696 31857433 PMC7456744

[B37] Han X. Lu H. Tang X. Zhao Y. Liu H. (2021). Immunogenomic characterization in gastric cancer identifies microenvironmental and immunotherapeutically relevant gene signatures. *Immun. Inflam. Dis.* 10 43–59. 10.1002/iid3.539 34582114 PMC8669697

[B38] Harjes U. (2020). Tumour-reactive T cells work remotely using IFNγ. *Nat. Rev. Cancer* 20:261. 10.1038/s41568-020-0255-0 32218523

[B39] Haura E. Turkson J. Jove R. (2005). Mechanisms of disease: Insights into the emerging role of signal transducers and activators of transcription in cancer. *Nat. Clin. Pract. Oncol.* 2 315–324. 10.1038/ncponc0195 16264989

[B40] Hellmann M. Callahan M. Awad M. Calvo E. Ascierto P. Atmaca A. (2018). Tumor mutational burden and efficacy of nivolumab monotherapy and in combination with ipilimumab in small-cell lung cancer. *Cancer cell* 33 853-61.e4. 10.1016/j.ccell.2018.04.001 29731394 PMC6750707

[B41] Hsieh Y. Tung S. Pan H. Yen C. Xu H. Lin Y. (2018). Increased abundance of clostridium and fusobacterium in gastric microbiota of patients with gastric cancer in taiwan. *Sci. Rep.* 8:158. 10.1038/s41598-017-18596-0 29317709 PMC5760541

[B42] Huang C. Li M. Liu B. Zhu H. Dai Q. Fan X. (2021). Relating gut microbiome and its modulating factors to immunotherapy in solid tumors: A systematic review. *Front. Oncol.* 11:642110. 10.3389/fonc.2021.642110 33816289 PMC8012896

[B43] Inamura K. (2021). Gut microbiota contributes towards immunomodulation against cancer: New frontiers in precision cancer therapeutics. *Semin. Cancer Biol.* 70 11–23. 10.1016/j.semcancer.2020.06.006 32580023

[B44] Ito K. Chuang L. Ito T. Chang T. Fukamachi H. Salto-Tellez M. (2011). Loss of Runx3 is a key event in inducing precancerous state of the stomach1. *Gastroenterology* 140 536-46.e8. 10.1053/j.gastro.2011.01.043 21277301

[B45] Ito K. Lim A. Salto-Tellez M. Motoda L. Osato M. Chuang L. (2008). RUNX3 attenuates beta-catenin/T cell factors in intestinal tumorigenesis. *Cancer Cell* 14 226–237. 10.1016/j.ccr.2008.08.004 18772112

[B46] Jones R. Mercante J. Neish A. (2012). Reactive oxygen production induced by the gut microbiota: pharmacotherapeutic implications. *Curr. Med. Chem.* 19 1519–1529. 10.2174/092986712799828283 22360484 PMC4269156

[B47] Kansy B. Concha-Benavente F. Srivastava R. Jie H. Shayan G. Lei Y. (2017). PD-1 Status in CD8 T cells associates with survival and Anti-PD-1 therapeutic outcomes in head and neck cancer. *Cancer Res.* 77 6353–6364. 10.1158/0008-5472.CAN-16-3167 28904066 PMC5690836

[B48] Koumarianou A. Krivan S. Machairas N. Ntavatzikos A. Pantazis N. Schizas D. (2019). Ten-year survival outcomes of patients with potentially resectable gastric cancer: impact of clinicopathologic and treatment-related risk factors. *Ann. Gastroenterol.* 32 99–106. 10.20524/aog.2018.0320 30598599 PMC6302201

[B49] Lazăr D. Avram M. Romoşan I. Cornianu M. Tăban S. Goldiş A. (2018). Prognostic significance of tumor immune microenvironment and immunotherapy: Novel insights and future perspectives in gastric cancer. *World J. Gastroenterol.* 24 3583–3616. 10.3748/wjg.v24.i32.3583 30166856 PMC6113718

[B50] Lefebvre O. Chenard M. Masson R. Linares J. Dierich A. LeMeur M. (1996). Gastric mucosa abnormalities and tumorigenesis in mice lacking the pS2 trefoil protein. *Science* 274 259–262. 10.1126/science.274.5285.259 8824193

[B51] Lertpiriyapong K. Whary M. Muthupalani S. Lofgren J. Gamazon E. Feng Y. (2014). Gastric colonisation with a restricted commensal microbiota replicates the promotion of neoplastic lesions by diverse intestinal microbiota in the Helicobacter pylori INS-GAS mouse model of gastric carcinogenesis. *Gut* 63 54–63. 10.1136/gutjnl-2013-305178 23812323 PMC4023484

[B52] Liang W. Yang Y. Wang H. Wang H. Yu X. Lu Y. (2019). Gut microbiota shifts in patients with gastric cancer in perioperative period. *Medicine* 98:e16626. 10.1097/MD.0000000000016626 31464899 PMC6736490

[B53] Ling Z. Shao L. Liu X. Cheng Y. Yan C. Mei Y. (2019). Regulatory T cells and plasmacytoid dendritic cells within the tumor microenvironment in gastric cancer are correlated with gastric microbiota Dysbiosis: a preliminary study. *Front. Immunol.* 10:533. 10.3389/fimmu.2019.00533 30936882 PMC6433099

[B54] Liou J. Chen C. Chang C. Fang Y. Bair M. Chen P. (2019). Long-term changes of gut microbiota, antibiotic resistance, and metabolic parameters after Helicobacter pylori eradication: a multicentre, open-label, randomised trial. *Lancet Infect. Dis.* 19 1109–1120. 10.1016/S1473-3099(19)30272-5 31559966

[B55] Lioux T. Mauny M. Lamoureux A. Bascoul N. Hays M. Vernejoul F. (2016). Design, Synthesis, and Biological Evaluation of Novel Cyclic Adenosine-Inosine Monophosphate (cAIMP) Analogs That Activate Stimulator of Interferon Genes (STING). *J. Med. Chem.* 59 10253–10267. 10.1021/acs.jmedchem.6b01300 27783523

[B56] Liu X. Shao L. Liu X. Ji F. Mei Y. Cheng Y. (2019). Alterations of gastric mucosal microbiota across different stomach microhabitats in a cohort of 276 patients with gastric cancer. *EBioMedicine* 40 336–348. 10.1016/j.ebiom.2018.12.034 30584008 PMC6412016

[B57] Lu Y. Yuan X. Wang M. He Z. Li H. Wang J. (2022). Gut microbiota influence immunotherapy responses: mechanisms and therapeutic strategies. *J. Hematol. Oncol.* 15:47. 10.1186/s13045-022-01273-9 35488243 PMC9052532

[B58] Marzagalli M. Ebelt N. Manuel E. (2019). Unraveling the crosstalk between melanoma and immune cells in the tumor microenvironment. *Semin. Cancer Biol.* 59 236–250. 10.1016/j.semcancer.2019.08.002 31404607

[B59] Matson V. Fessler J. Bao R. Chongsuwat T. Zha Y. Alegre M. (2018). The commensal microbiome is associated with anti-PD-1 efficacy in metastatic melanoma patients. *Science* 359 104–108. 10.1126/science.aao3290 29302014 PMC6707353

[B60] Matsuzaki J. Tsugawa H. Suzuki H. (2021). Precision Medicine Approaches to Prevent Gastric Cancer. *Gut Liver* 15 3–12. 10.5009/gnl19257 31893631 PMC7817924

[B61] Matthews G. Howarth G. Butler R. (2007). Short-chain fatty acid modulation of apoptosis in the Kato III human gastric carcinoma cell line. *Cancer Biol. Ther.* 6 1051–1057. 10.4161/cbt.6.7.4318 17611404

[B62] McCracken K. Catá E. Crawford C. Sinagoga K. Schumacher M. Rockich B. (2014). Modelling human development and disease in pluripotent stem-cell-derived gastric organoids. *Nature* 516 400–404. 10.1038/nature13863 25363776 PMC4270898

[B63] McQuade J. Daniel C. Helmink B. Wargo J. (2019). Modulating the microbiome to improve therapeutic response in cancer. *Lancet Oncol.* 20 e77–e91. 10.1016/S1470-2045(18)30952-5 30712808 PMC12908161

[B64] Michaud D. (2013). Role of bacterial infections in pancreatic cancer. *Carcinogenesis* 34 2193–2197. 10.1093/carcin/bgt249 23843038 PMC3786384

[B65] Motzer R. Escudier B. McDermott D. George S. Hammers H. Srinivas S. (2015). Nivolumab versus Everolimus in Advanced Renal-Cell Carcinoma. *N. Engl. J. Med.* 373 1803–1813. 10.1056/NEJMoa1510665 26406148 PMC5719487

[B66] Naseem M. Barzi A. Brezden-Masley C. Puccini A. Berger M. Tokunaga R. (2018). Outlooks on Epstein-Barr virus associated gastric cancer. *Cancer Treat. Rev.* 66 15–22. 10.1016/j.ctrv.2018.03.006 29631196 PMC5964025

[B67] Nie S. Wang A. Yuan Y. (2021). Comparison of clinicopathological parameters, prognosis, micro-ecological environment and metabolic function of Gastric Cancer with or without *Fusobacterium* sp. Infection. *J. Cancer* 12 1023–1032. 10.7150/jca.50918 33442401 PMC7797643

[B68] Nishikawa K. Murotani K. Fujitani K. Inagaki H. Akamaru Y. Tokunaga S. (1990). Differences in disease status between patients with progression after first-line chemotherapy versus early relapse after adjuvant chemotherapy who undergo second-line chemotherapy for gastric cancer: Exploratory analysis of the randomized phase III TRICS trial. *Eur. J. Cancer* 2020 159–167. 10.1016/j.ejca.2020.03.027 32380427

[B69] Nishikawa K. Murotani K. Fujitani K. Inagaki H. Akamaru Y. Tokunaga S. (2019). A study of second-line irinotecan plus cisplatin vs. irinotecan alone in platinum-naïve patients with early relapse of gastric cancer refractory to adjuvant S-1 monotherapy: exploratory subgroup analysis of the randomized phase III TRICS trial. *Cancer Chemother. Pharmacol.* 83 867–874. 10.1007/s00280-019-03802-9 30806758

[B70] Oguma K. Oshima H. Aoki M. Uchio R. Naka K. Nakamura S. (2008). Activated macrophages promote Wnt signalling through tumour necrosis factor-alpha in gastric tumour cells. *EMBO J.* 27 1671–1681. 10.1038/emboj.2008.105 18511911 PMC2413189

[B71] Oh S. Sohn B. Cheong J. Kim S. Lee J. Park K. (2018). Clinical and genomic landscape of gastric cancer with a mesenchymal phenotype. *Nat. Commun.* 9:1777. 10.1038/s41467-018-04179-8 29725014 PMC5934392

[B72] Olnes M. Martinson H. (2021). Recent advances in immune therapies for gastric cancer. *Cancer Gene Ther.* 28 924–934. 10.1038/s41417-021-00310-y 33664460 PMC8417143

[B73] Orchel A. Dzierzewicz Z. Parfiniewicz B. Weglarz L. Wilczok T. (2005). Butyrate-induced differentiation of colon cancer cells is PKC and JNK dependent. *Digest. Dis. Sci.* 50 490–498. 10.1007/s10620-005-2463-6 15810631

[B74] Oshima H. Hioki K. Popivanova B. Oguma K. Van Rooijen N. Ishikawa T. (2011). Prostaglandin E2 signaling and bacterial infection recruit tumor-promoting macrophages to mouse gastric tumors. *Gastroenterology* 140 596-607.e7. 10.1053/j.gastro.2010.11.007 21070778

[B75] Oster P. Vaillant L. McMillan B. Velin D. (2022). *Helicobacter pylori* The efficacy of cancer immunotherapies is compromised by infection. *Front. Immunol.* 13:899161. 10.3389/fimmu.2022.899161 35677057 PMC9168074

[B76] Oster P. Vaillant L. Riva E. McMillan B. Begka C. Truntzer C. (2021). Helicobacter pylori infection has a detrimental impact on the efficacy of cancer immunotherapies. *Gut* 71 457–466. 10.1136/gutjnl-2020-323392 34253574 PMC8862014

[B77] Park W. Oh R. Park J. Lee J. Shin M. Kim H. (2000). Somatic mutations of the trefoil factor family 1 gene in gastric cancer. *Gastroenterology* 119 691–698. 10.1053/gast.2000.16483 10982763

[B78] Peng C. Ouyang Y. Lu N. Li N. (2020). The NF-κB signaling pathway, the microbiota, and gastrointestinal tumorigenesis: recent advances. *Front. Immunol.* 11:1387. 10.3389/fimmu.2020.01387 32695120 PMC7338561

[B79] Peng Z. Cheng S. Kou Y. Wang Z. Jin R. Hu H. (2020). The Gut Microbiome Is Associated with Clinical Response to Anti-PD-1/PD-L1 Immunotherapy in Gastrointestinal Cancer. *Cancer Immunol. Res.* 8 1251–1261. 10.1158/2326-6066.CIR-19-1014 32855157

[B80] Pereira-Marques J. Ferreira R. Machado J. Figueiredo C. (2021). The influence of the gastric microbiota in gastric cancer development. *Best Pract. Res. Clin. Gastroenterol.* 2021:101734. 10.1016/j.bpg.2021.101734 33975676

[B81] Pereira-Marques J. Ferreira R. Pinto-Ribeiro I. Figueiredo C. (2019). Helicobacter pylori infection, the gastric microbiome and gastric cancer. *Adv. Exp. Med. Biol.* 1149 195–210. 10.1007/5584_2019_366 31016631

[B82] Pharoah P. Guilford P. Caldas C. (2001). Incidence of gastric cancer and breast cancer in CDH1 (E-cadherin) mutation carriers from hereditary diffuse gastric cancer families. *Gastroenterology* 121 1348–1353. 10.1053/gast.2001.29611 11729114

[B83] Pianta A. Arvikar S. Strle K. Drouin E. Wang Q. Costello C. (2017). Evidence of the Immune Relevance of Prevotella copri, a Gut Microbe, in Patients With Rheumatoid Arthritis. *Arthr. Rheumatol.* 69 964–975. 10.1002/art.40003 27863183 PMC5406252

[B84] Qiu Q. Lin Y. Ma Y. Li X. Liang J. Chen Z. (2021). Exploring the emerging role of the gut microbiota and tumor microenvironment in cancer immunotherapy. *Front. Immunol.* 11:612202. 10.3389/fimmu.2020.612202 33488618 PMC7817884

[B85] Ratajczak W. Rył A. Mizerski A. Walczakiewicz K. Sipak O. Laszczyñska M. (2019). Immunomodulatory potential of gut microbiome-derived short-chain fatty acids (SCFAs). *Acta Biochim. Polonica* 66 1–12. 10.18388/abp.2018_2648 30831575

[B86] Ren F. Zhao Q. Zhao M. Zhu S. Liu B. Bukhari I. (2021). Immune infiltration profiling in gastric cancer and their clinical implications. *Cancer Sci.* 112 3569–3584. 10.1111/cas.15057 34251747 PMC8409427

[B87] Ribas A. Wolchok J. (2018). Cancer immunotherapy using checkpoint blockade. *Science* 359 1350–1355. 10.1126/science.aar4060 29567705 PMC7391259

[B88] Routy B. Le Chatelier E. Derosa L. Duong C. P. M. Alou M. T. Daillère R. (2018). Gut microbiome influences efficacy of PD-1-based immunotherapy against epithelial tumors. *Science* 359 91–97. 10.1126/science.aan3706 29097494

[B89] Sarhadi V. Mathew B. Kokkola A. Karla T. Tikkanen M. Rautelin H. (2021). Gut microbiota of patients with different subtypes of gastric cancer and gastrointestinal stromal tumors. *Gut Pathog.* 13:11. 10.1186/s13099-021-00403-x 33596997 PMC7888145

[B90] Scher J. Sczesnak A. Longman R. Segata N. Ubeda C. Bielski C. (2013). Expansion of intestinal Prevotella copri correlates with enhanced susceptibility to arthritis. *eLife* 2:e01202. 10.7554/eLife.01202.028 24192039 PMC3816614

[B91] Shinohara Y. Tsukimoto M. (2018). Guanine and inosine nucleotides/nucleosides suppress murine T cell activation. *Biochem. Biophy. Res. Commun.* 498 764–768. 10.1016/j.bbrc.2018.03.055 29524424

[B92] Singh V. Yang J. Chen T. Zachos N. Kovbasnjuk O. Verkman A. (2014). Translating molecular physiology of intestinal transport into pharmacologic treatment of diarrhea: stimulation of Na+ absorption. *Clin. Gastroenterol. Hepatol.* 12 27–31. 10.1016/j.cgh.2013.10.020 24184676 PMC3926754

[B93] Sivan A. Corrales L. Hubert N. Williams J. Aquino-Michaels K. Earley Z. (2015). Commensal Bifidobacterium promotes antitumor immunity and facilitates anti-PD-L1 efficacy. *Science* 350 1084–1089. 10.1126/science.aac4255 26541606 PMC4873287

[B94] Smet A. Kupcinskas J. Link A. Hold G. Bornschein J. (2021). The role of microbiota in gastrointestinal cancer and cancer treatment - chance or curse?. *Cell. Mol. Gastroenterol. Hepatol.* 13 857–874. 10.1016/j.jcmgh.2021.08.013 34506954 PMC8803618

[B95] Song W. Anselmo A. Huang L. (2019). Nanotechnology intervention of the microbiome for cancer therapy. *Nat. Nanotechnol.* 14 1093–1103. 10.1038/s41565-019-0589-5 31802032

[B96] Song X. Xin N. Wang W. Zhao C. (2015). Wnt/β-catenin, an oncogenic pathway targeted by H. pylori in gastric carcinogenesis. *Oncotarget* 6 35579–35588. 10.18632/oncotarget.5758 26417932 PMC4742126

[B97] Sun J. Li X. Yin J. Li Y. Hou B. Zhang Z. (2018). A screening method for gastric cancer by oral microbiome detection. *Oncol. Rep.* 39 2217–2224. 10.3892/or.2018.6286 29498406

[B98] Sun J. Tang Q. Yu S. Xie M. Xie Y. Chen G. (2020). Role of the oral microbiota in cancer evolution and progression. *Cancer Med.* 9 6306–6321. 10.1002/cam4.3206 32638533 PMC7476822

[B99] Sun J. Zhou M. Salazar C. Hays R. Bedi S. Chen Y. (2017). Chronic periodontal disease, periodontal pathogen colonization, and increased risk of precancerous gastric lesions. *J. Periodontol.* 88 1124–1134. 10.1902/jop.2017.160829 28671506

[B100] Sung H. Ferlay J. Siegel R. L. Laversanne M. Soerjomataram I. Jemal A. (2021). Global cancer statistics 2020: Globocan estimates of incidence and mortality worldwide for 36 cancers in 185 countries. *CA Cancer J. Clin.* 71, 209–249. 10.3322/caac.21660 33538338

[B101] Suzuki M. Mimuro H. Kiga K. Fukumatsu M. Ishijima N. Morikawa H. (2009). Helicobacter pylori CagA phosphorylation-independent function in epithelial proliferation and inflammation. *Cell Host Microbe* 5 23–34. 10.1016/j.chom.2008.11.010 19154985

[B102] Szkaradkiewicz A. K. Karpinski T. (2013). Microbiology of chronic periodontitis. *J. Biol. Earth Sci.* 3 14–20.

[B103] The Cancer Genome Atlas Research Network (2014). Comprehensive molecular characterization of gastric adenocarcinoma. *Nature* 513 202–209. 10.1038/nature13480 25079317 PMC4170219

[B104] Tomita H. Takaishi S. Menheniott T. Yang X. Shibata W. Jin G. (2011). Inhibition of gastric carcinogenesis by the hormone gastrin is mediated by suppression of TFF1 epigenetic silencing. *Gastroenterology* 140 879–891. 10.1053/j.gastro.2010.11.037 21111741 PMC3049860

[B105] Tsang Y. Lamb A. Romero-Gallo J. Huang B. Ito K. Peek R. (2010). Helicobacter pylori CagA targets gastric tumor suppressor RUNX3 for proteasome-mediated degradation. *Oncogene* 29 5643–5650. 10.1038/onc.2010.304 20676134 PMC2980823

[B106] Tseng C. Lin J. Ho H. Lai Z. Wang C. Tang S. (2016). Gastric microbiota and predicted gene functions are altered after subtotal gastrectomy in patients with gastric cancer. *Sci. Rep.* 6:20701. 10.1038/srep20701 26860194 PMC4748256

[B107] Tuominen H. Rautava J. (2021). Oral microbiota and cancer development. *Pathobiology* 88 116–126. 10.1159/000510979 33176328

[B108] Vétizou M. Pitt J. Daillère R. Lepage P. Waldschmitt N. Flament C. (2015). Anticancer immunotherapy by CTLA-4 blockade relies on the gut microbiota. *Science* 350 1079–1084. 10.1126/science.aad1329 26541610 PMC4721659

[B109] Vogelmann R. Amieva M. (2007). The role of bacterial pathogens in cancer. *Curr. Opin. Microbiol.* 10 76–81. 10.1016/j.mib.2006.12.004 17208515

[B110] Wang D. Li Y. Zhong H. Ding Q. Lin Y. Tang S. (2019). Alterations in the human gut microbiome associated with Helicobacter pylori infection. *FEBS Open Bio* 9 1552–1560. 10.1002/2211-5463.12694 31250988 PMC6724102

[B111] Wang D. Zhang T. Lu Y. Wang C. Wu Y. Li J. (2022). Helicobacter pylori infection affects the human gastric microbiome, as revealed by metagenomic sequencing. *FEBS Open Bio* 12 1188–1196. 10.1002/2211-5463.13390 35243810 PMC9157398

[B112] Wang F. Wu J. Wang Y. Jin Y. Jiang X. Qiu Z. (2019). Gut microbiota functional biomolecules with immune-lipid metabolism for a prognostic compound score in epstein-barr virus-associated gastric adenocarcinoma: a pilot study. *Clin. Trans. Gastroenterol.* 10:e00074. 10.14309/ctg.0000000000000074 31609743 PMC6884346

[B113] Wang L. Xin Y. Zhou J. Tian Z. Liu C. Yu X. (2020). Gastric mucosa-associated microbial signatures of early gastric cancer. *Front. Microbiol.* 11:1548. 10.3389/fmicb.2020.01548 32733423 PMC7358557

[B114] Wang T. Gnanaprakasam J. Chen X. Kang S. Xu X. Sun H. (2020). Inosine is an alternative carbon source for CD8-T-cell function under glucose restriction. *Nat. Metab.* 2 635–647. 10.1038/s42255-020-0219-4 32694789 PMC7371628

[B115] Wang Y. Wiesnoski D. Helmink B. Gopalakrishnan V. Choi K. DuPont H. (2018). Fecal microbiota transplantation for refractory immune checkpoint inhibitor-associated colitis. *Nat. Med.* 24 1804–1808. 10.1038/s41591-018-0238-9 30420754 PMC6322556

[B116] Wang Z. Gao X. Zeng R. Wu Q. Sun H. Wu W. (2020). Changes of the gastric mucosal microbiome associated with histological stages of gastric carcinogenesis. *Front. Microbiol.* 11:997. 10.3389/fmicb.2020.00997 32547510 PMC7272699

[B117] Weber J. Mandala M. Del Vecchio M. Gogas H. Arance A. Cowey C. (2017). Adjuvant Nivolumab versus Ipilimumab in Resected Stage III or IV Melanoma. *N. Engl. J. Med.* 377 1824–1835. 10.1056/NEJMoa1709030 28891423

[B118] Weng M. Chiu Y. Wei P. Chiang C. Fang H. Wei S. (2019). Microbiota and gastrointestinal cancer. *J. Formosan Med. Assoc.* 2019 S32–S41. 10.1016/j.jfma.2019.01.002 30655033

[B119] Wu J. Xu S. Xiang C. Cao Q. Li Q. Huang J. (2018). Tongue coating microbiota community and risk effect on gastric cancer. *J. Cancer* 9 4039–4048. 10.7150/jca.25280 30410609 PMC6218773

[B120] Wu Z. Zou K. Wu G. Jin Z. Xiang C. Xu S. (2021). A comparison of tumor-associated and non-tumor-associated gastric microbiota in gastric cancer patients. *Digest. Dis. Sci.* 66 1673–1682. 10.1007/s10620-020-06415-y 32591968

[B121] Xavier J. Young V. Skufca J. Ginty F. Testerman T. Pearson A. (2020). The cancer microbiome: Distinguishing direct and indirect effects requires a systemic view. *Trends Cancer* 6 192–204. 10.1016/j.trecan.2020.01.004 32101723 PMC7098063

[B122] Xu J. Liu M. Tao T. Zhu X. Fei F. (2021). The role of gut microbiota in tumorigenesis and treatment. *Biomed. Pharmacother.* 138:111444. 10.1016/j.biopha.2021.111444 33662679

[B123] Yan F. Cao H. Chaturvedi R. Krishna U. Hobbs S. Dempsey P. (2009). Epidermal growth factor receptor activation protects gastric epithelial cells from Helicobacter pylori-induced apoptosis. *Gastroenterology* 136 1297–1307, e1-3. 10.1053/j.gastro.2008.12.059 19250983 PMC2878739

[B124] Yang P. Zhou Y. Chen B. Wan H. Jia G. Bai H. (1990). Overweight, obesity and gastric cancer risk: results from a meta-analysis of cohort studies. *Eur. J. Cancer* 2009 2867–2873. 10.1016/j.ejca.2009.04.019 19427197

[B125] Yang Y. Huang Y. Lin W. Liu J. Chen X. Chen C. (2022a). Host miRNAs-microbiota interactions in gastric cancer. *J. Trans. Med.* 20:52. 10.1186/s12967-022-03264-3 35093110 PMC8800214

[B126] Yang Y. Long J. Wang C. Blot W. Pei Z. Shu X. (2022b). Prospective study of oral microbiome and gastric cancer risk among Asian, African American and European American populations. *Int. J. Cancer* 150 916–927. 10.1002/ijc.33847 34664266 PMC8982516

[B127] Yap T. Gan H. Lee Y. Leow A. Azmi A. Francois F. (2016). Helicobacter pylori eradication causes perturbation of the human gut microbiome in young adults. *PLoS One* 11:e0151893. 10.1371/journal.pone.0151893 26991500 PMC4798770

[B128] Yu G. Torres J. Hu N. Medrano-Guzman R. Herrera-Goepfert R. Humphrys M. (2017). Molecular characterization of the human stomach microbiota in gastric cancer patients. *Front. Cell. Infect. Microbiol.* 7:302. 10.3389/fcimb.2017.00302 28730144 PMC5498480

[B129] Zhang X. Zhang D. Jia H. Feng Q. Wang D. Liang D. (2015). The oral and gut microbiomes are perturbed in rheumatoid arthritis and partly normalized after treatment. *Nat. Med.* 21 895–905. 10.1038/nm.3914 26214836

[B130] Zhang Y. Shen J. Shi X. Du Y. Niu Y. Jin G. (2021). Gut microbiome analysis as a predictive marker for the gastric cancer patients. *Appl. Microbiol. Biotechnol.* 105 803–814. 10.1007/s00253-020-11043-7 33404833

[B131] Zhang Z. Feng H. Qiu Y. Xu Z. Xie Q. Ding W. (2022a). Dysbiosis of gastric mucosal fungal microbiota in the gastric cancer microenvironment. *J. Immunol. Res.* 2022:6011632. 10.1155/2022/6011632 35340583 PMC8942701

[B132] Zhang Z. Zhu L. Ma Y. Wang B. Ci C. Zhang J. (2022b). Study on the characteristics of intestinal flora composition in gastric cancer patients and healthy people in the qinghai-tibet plateau. *Appl. Biochem. Biotechnol.* 194 1510–1526. 10.1007/s12010-021-03732-4 34792749 PMC9007807

[B133] Zhong M. Xiong Y. Zhao J. Gao Z. Ma J. Wu Z. (2021). Candida albicans disorder is associated with gastric carcinogenesis. *Theranostics* 11 4945–4956. 10.7150/thno.55209 33754037 PMC7978306

[B134] Zitvogel L. Ma Y. Raoult D. Kroemer G. Gajewski T. (2018). The microbiome in cancer immunotherapy: Diagnostic tools and therapeutic strategies. *Science* 359 1366–1370. 10.1126/science.aar6918 29567708

